# Maternal β-Cell Adaptations in Pregnancy and Placental Signalling: Implications for Gestational Diabetes

**DOI:** 10.3390/ijms19113467

**Published:** 2018-11-05

**Authors:** Brittany L. Moyce, Vernon W. Dolinsky

**Affiliations:** Department of Pharmacology & Therapeutics and the Diabetes Research Envisioned and Accomplished in Manitoba (DREAM) Research Theme of the Children’s Hospital Research Institute of Manitoba and the Manitoba Developmental Origins of Chronic Diseases in Children Network (DEVOTION), University of Manitoba, Winnipeg, MB R3E 3P4, Canada; ummoyce@myumanitoba.ca

**Keywords:** gestational diabetes mellitus, β-cell, insulin, pregnancy, placenta

## Abstract

Rates of gestational diabetes mellitus (GDM) are on the rise worldwide, and the number of pregnancies impacted by GDM and resulting complications are also increasing. Pregnancy is a period of unique metabolic plasticity, during which mild insulin resistance is a physiological adaptation to prioritize fetal growth. To compensate for this, the pancreatic β-cell utilizes a variety of adaptive mechanisms, including increasing mass, number and insulin-secretory capacity to maintain glucose homeostasis. When insufficient insulin production does not overcome insulin resistance, hyperglycemia can occur. Changes in the maternal system that occur in GDM such as lipotoxicity, inflammation and oxidative stress, as well as impairments in adipokine and placental signalling, are associated with impaired β-cell adaptation. Understanding these pathways, as well as mechanisms of β-cell dysfunction in pregnancy, can identify novel therapeutic targets beyond diet and lifestyle interventions, insulin and antihyperglycemic agents currently used for treating GDM.

## 1. Introduction

Gestational diabetes mellitus (GDM) is defined as hyperglycemia and severe insulin resistance with an onset in mid-gestation near the beginning of the 3rd trimester. The worldwide incidence of pregnancies impacted by gestational diabetes mellitus (GDM) is as high as 1 in 7 [1]. Health Canada statistics from 2011 place the rate of GDM at roughly 5.4%, which is only increasing as more women enter pregnancy obese or overweight. In Canada, as well as many Western nations, more women are becoming pregnant later in life, increasing their risk for GDM [2,3]. The implications of this trend are potentially wide reaching—complications of GDM can include difficulties with pregnancy itself, labor and delivery complications, and maternal health implications postnatally and onward [1,4,5]. Additionally, research has implicated fetal exposure to GDM in development of respiratory distress syndrome, pre-term birth, and higher incidence of metabolic and cardiac dysfunction later in life [6,7]. In this review, we describe the placental signalling and maternal β-cell adaptations that occur during pregnancy and consider how maladaptations in these processes contribute to the development of GDM.

## 2. Metabolic Adaptations during Mammalian Pregnancy

Mammalian pregnancy is marked by distinct phases of metabolic adaptation, correlating to changing nutritional needs of both the mother and the fetus. Early pregnancy (in humans characterized as the first two trimesters; in rodents, the first 10 days of gestation) is a largely anabolic phase, during which energy stores are built up in the form of lipid deposition in tissues, for breakdown and utilization in late gestation [8,9]. This is achieved by increases in maternal energy consumption as well as increased de novo lipogenesis by the liver. In late gestation, these lipid deposits are preferentially broken down for maternal use, and in turn glucose is spared to meet the increasing energy demands of the growing fetus [8,9,10]. This later stage of pregnancy is referred to as the catabolic phase. Pregnancy is by necessity a period of relative metabolic plasticity, during which physiological changes need to occur to accommodate shifting nutritional needs. These adaptations are achieved through a variety of mechanisms, including hormonal, metabolic and immunological alterations [9]. Many maternal systems will undergo adaptations to accommodate pregnancy, including increases in blood volume and cardiac output with corresponding increases in renal activity, increased respiratory capacity and neurological changes [11].

**Figure 1:**
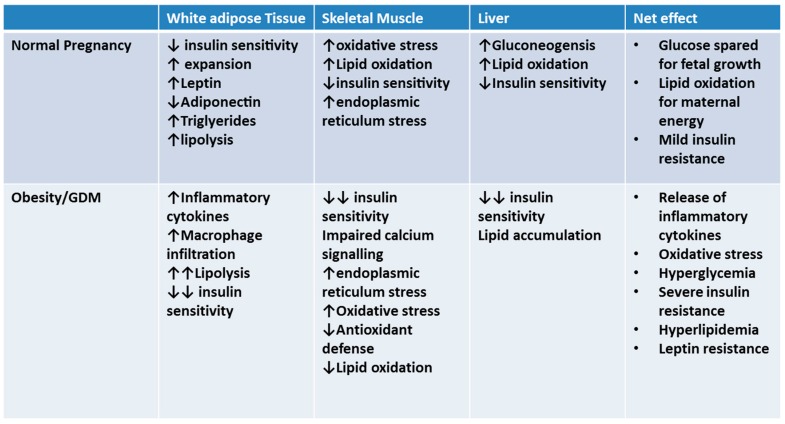
Adaptations in peripheral tissue in late gestation occur to spare glucose for fetal growth; if insulin resistance is too severe (such as in pregnancies complicated by pre-existing obesity), gestational diabetes can occur.

As the maternal system enters the catabolic phase in late gestation, insulin resistance and hyperlipidemia are a natural, albeit transient occurrence (Figure 1). Despite these changes, maternal blood glucose remains constant or, indeed, has been shown to decrease as pregnancy progresses; while this may be due in part to dilution due to increased blood volume, the major driver is an increase in maternal insulin production and secretion by the pancreas [12]. Since peripheral tissues become more insulin resistant [9,13] in pregnancy and hepatic glucose production and output continues (Figure 1), glucose homeostasis is achieved in a healthy pregnancy by increasing circulating insulin and overcoming insulin resistance [8]. 

In the context of these metabolic and β-cell adaptations during pregnancy, GDM may be viewed as an inability to compensate for the insulin resistance of pregnancy. In the presence of pre-existing maternal obesity or excessive gestational weight gain results, insulin secretion is insufficient to overcome insulin resistance and maintain glucose homeostasis, resulting in hyperglycemia and glucose intolerance that is characteristic of GDM [14].

While many studies exist which extensively characterize and summarize the adaptive response of the pancreatic β-cell to the metabolic stress of pregnancy, there is less information available regarding defects in these adaptive mechanisms. Understanding where and how β-cell dysfunction occurs (particularly during pregnancy) will help to determine why some pregnancies are more susceptible to the additive effects of obesity, and contribute to defining the etiology of GDM and the development of new therapeutic approaches.

## 3. β-Cell Adaptations

In pregnancy, compensatory increases in β-cell mass are achieved through a combination of hypertrophic expansion, proliferation and potentially neogenesis from precursor cells, accompanied by a temporary decrease in apoptosis [12,15,16]. These adaptations have been well characterized in human pregnancy [12]. Mechanisms have been investigated with the use of animal models illustrating the necessary crosstalk between the maternal pancreas, placenta, and peripheral tissues [17,18,19]. While many of these mechanisms are conserved, there are some differences between human and rodent adaptive pathways—for example, most studies in rodent models describe small increases in β-cell neogenesis (if any at all) [20], but in human pregnancy, neogenesis may have a larger role than proliferation [16,21]. It is important to keep these mechanistic differences in mind, particularly when translating research from rodents to human populations—however, the relative scarcity of human islets in pregnancy, and the heterogeneity of the samples that do exist present challenges in their own right [12].

## 4. Pregnancy Hormones: Prolactin and Placental Lactogen

Expansion of maternal insulin secretion capacity is a central adaptation required to maintain a metabolically healthy pregnancy. While adult β-cell populations are known to adapt to a variety of physiological conditions, pregnancy involves β-cell adaptations [10], which occur via functional changes (insulin production and secretion) and morphological changes such as proliferation and expansion [22,23]. It has been noted that these adaptations in the β-cell occur prior to the onset of insulin resistance in pregnancy, and actually occur in response to pregnancy itself. Studies have identified many genes with postulated roles in mediating these changes [10,23,24,25]; they are primarily downstream of the lactogens. Signalling occurs largely through placental lactogen (PL) and prolactin (PrL), but hepatic growth factor (HGF), progestin and estrogen are also implicated in both adaptive increases in β-cell mass and function, as well as the return to baseline postnatally [15,26]. HGF expression increases in pregnancy and signals through c-met and the AKT cascade to facilitate β-cell adaptations [15,27]. The absence of HGF signalling impacts glucose sensing, when cells that lack HGF are unable to upregulate GLUT2 [26].

**Figure 2:**
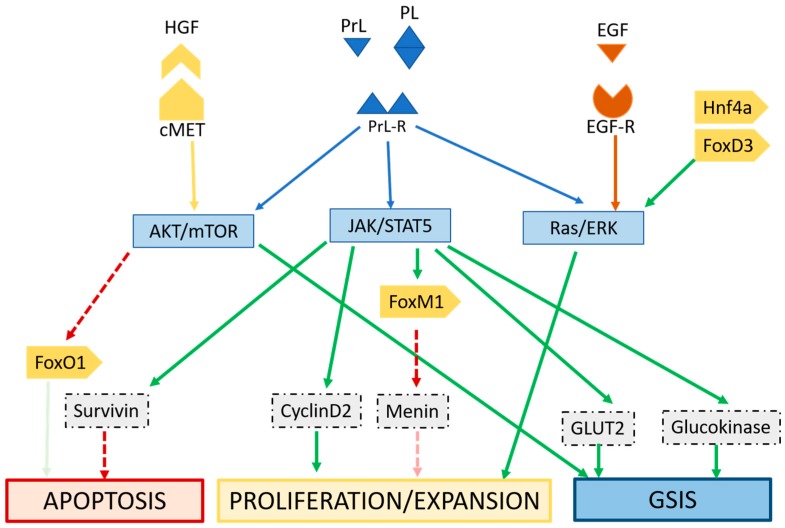
Signalling by pregnancy hormones prolactin (PrL) and placental lactogen (PL) through the prolactin receptor (PrlR) through the Jak/Stat pathway improves glucose stimulated insulin secretion (GSIS), prevents apoptosis and promotes proliferation and expansion. Hepatocyte growth factor (HGF) signals through AKT and mTOR via cMet to improve β-cell adaptations. Epidermal growth factor (EGF) through epidermal growth factor receptor (EGF-R) and ras/extracellular signal-related kinases (ERK) signalling can mediate proliferation and expansion; the ras/ERK pathway can also be mediated lactogens. Transcription factors (such as Hnf4a, FoxD3, FoxM1) can modulate expression of genes that mediate these adaptations.

Mechanistic studies in rodent models [10,25,28,29,30] and some limited work in human islets—[12,31] have identified key pathways responsible for mediating PL signalling (Figure 2). Within the endocrine pancreas, genes responsible for synthesis of serotonin are upregulated by PL signalling. Interruption of this signalling has been shown to impair the proliferation of β-cells required in pregnancy [7,15]. Serotonin signalling also plays an important role in glucose stimulated insulin secretion in pregnancy [28], maintaining glucose homeostasis and sensitivity. If serotonin signalling downstream of PL is disrupted, compensatory β-cell proliferation and insulin secretion during pregnancy is impaired [7,32].

In addition, serotonin signalling from the β-cell (triggered by metabolic stress such as high fat diet or pregnancy) inhibits glucagon secretion by the α-cell [33]. Impairments in serotonin sensing or reduced levels of serotonin can impede the ability of the α-cell to regulate glucagon secretion in response to blood glucose; this effect on glucagon secretion can compound with β-cell impairments and worsen metabolic adaptation to pregnancy [33]. 

Epidermal growth factor (EGF) also acts at the β-cell, through EGF-R and improves proliferation and expansion [29] (Figure 2). Interestingly, vascular endothelial growth factor (VEGF) originating from pancreatic endothelium has been shown to impact signalling of HGF [27], highlighting the importance of endothelial-endocrine crosstalk within the placenta. 

## 5. β-Cell Pathology in GDM

Throughout gestation, maternal metabolism prioritizes glucose and nutrient availability for fetal growth. The resultant insulin resistance is transient in a healthy pregnancy. Pre-existing obesity, maternal over-nutrition, excessive gestational weight gain, as well as certain genetic predispositions can trigger failure of the β-cell and hyperglycemia. β-cell adaptations are carefully coordinated during pregnancy. If one or more pathways are impaired, the failure to compensate for the insulin resistance of pregnancy can lead to hyperglycemia and GDM. Indeed, β-cell failure or insufficiency has been implicated in type 2 diabetes, and auto-immune mediated attack on β-cells is a driver of β-cell loss in type 1 diabetes [15]. Recent research has defined several mechanisms of β-cell adaptation to pregnancy (as reviewed by Ernst et al. [15] and Baeyens et al. [34]); however, the mechanisms underlying β-cell dysfunction in pregnancy are less clear. Existing studies in human islets during pregnancy vary widely with respect to maternal BMI and gestational age. Additionally, none of the human studies include samples from mothers with GDM, which could provide insight into mechanisms of adaptive failure by the β-cell in GDM.

Mutations that affect islet function are linked to subsets of diabetes known as maturity onset diabetes of the young (MODY), but relatively few mutations in transcription factors with roles in β-cell function and adaptation (such as *hnf1a*, *foxd3*, *foxM1*, *hnf4a*) have been implicated in development of GDM [23,34]. An additional level of control is introduced with the action of micro-RNAs, which regulate gene expression and impact adaptive mechanisms. Micro RNA (miRNA) associated with β-cell plasticity include miR375, which increases β-cell expansion, and miR7a that increased signalling through the mammalian target of rapamycin (mTOR) (Figure 2) and various miRNAs activate neurogenin-3 and in doing so increase neogenesis [35]. Downregulation of miR338-3p [36] is postulated to potentiate the actions of incretins, specifically glucagon-like peptide (GLP)-1, which enhances insulin secretion and proliferation in β-cells, and sensitivity in peripheral tissues [37].

## 6. Oxidative Stress and Inflammation

Oxidative stress, mitochondrial dysfunction and endoplasmic reticulum (ER) stress are observed in insulin-resistant peripheral tissues in GDM [38,39]. While the inflammatory profile of pregnancy is dynamic, disruptions in inflammatory cytokine profiles and macrophage infiltration of insulin sensitive tissues (such as white adipose tissue) can contribute to worsening insulin resistance thereby contributing to the development of GDM [14,40]. Similarly, the β-cell is also susceptible to these types of dysfunction: in addition to insulin sensitivity, the β-cell is also impacted by systemic inflammation [13]. Individuals with obesity or GDM have higher levels of pro-inflammatory cytokine tumor necrosis factor α (TNF-α) in the circulation, which is linked to impaired β-cell function as well as β-cell de-differentiation [40,41]. Additional inflammatory markers such as interleukin-1β (IL-1β) and interferon-γ (IFNγ) have been shown to be elevated in the presence of metabolic stress [38,39], and these can trigger ER stress in the β-cell, ultimately leading to β-cell dysfunction [42].

In type 2 diabetes, β-cell de-differentiation can be triggered by inflammatory cytokines [41]. Indeed, β-cells can be susceptible to macrophage infiltration as well [42] and inhibition of factors relating to macrophage recruitment have been shown to improve β-cell function [43]. This can be especially detrimental, as the maternal system shifts back towards a more “pro-inflammatory” immune state in late gestation, when insulin resistance is at its peak and GDM can occur [40].

## 7. Lipotoxicity and Oxidative Stress

In general, nutrient overload in obesity (and is worsened by peripheral insulin resistance) exerts a multitude of effects on the β-cell. Pancreatic β-cells are susceptible to lipotoxicity, and prolonged exposure to elevated lipids can trigger β-cell dysfunction. Lipotoxicity and glucotoxicity are possible mechanisms underlying β-cell dysfunction in type 2 diabetes and GDM [43]. The resulting buildup of lipids in the pancreatic islet can cause ER stress [44] and oxidative stress [43], which impair insulin production. These mechanisms ultimately contribute to β-cell apoptosis [43].

**Figure 3:**
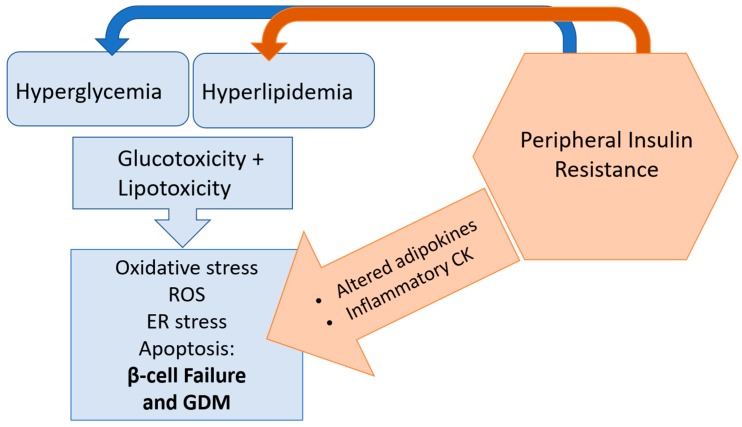
Peripheral insulin resistance and resulting hyperlipidemia and hyperglycemia can overwhelm the capacity of the β-cell, leading to lipotoxicity and glucotoxicity. Lipotoxicity and glucotoxicity can trigger oxidative stress, generation of reactive oxygen species (ROS) and lead to endoplasmic reticulum (ER) stress and apoptosis; these detrimental pathways impair β-cell adaptation in pregnancy and can lead to gestational diabetes mellitus (GDM).

Metabolomic studies of pregnant women with GDM and a mouse model of high fat diet feeding during pregnancy identified 3-carboxy-4-methyl-5-pentyl-2-furanpropionic acid (CMPF), a furan fatty acid metabolite as having a causative role in the mitochondrial dysfunction and resulting oxidative stress that can impair compensatory β-cell function during pregnancy [45]. Mitochondrial dysfunction can result from hyperlipidemia and prolonged exposure to metabolic stress [14,45]; expression of UCP2, an uncoupling protein, has been documented in cases of β-cell dysfunction—further driving the production of reactive oxygen species, reducing ATP production and decreasing insulin output [46,47]. In addition, the β-cell can become overwhelmed by prolonged exposure to hyperglycemia; the resulting glucotoxicity generates oxidative stress within the β-cell, leading to decreased survival and reduced glucose stimulated insulin secretion. If signals such as inflammation and nutrient mediated toxicity continue to overwhelm the β-cell, apoptosis and β-cell death can occur (Figure 3) [48].

## 8. Adipokine Signalling

Cytokines released from adipose tissue (adipokines) have roles in metabolism that remain important during pregnancy. Leptin is well characterized in metabolism and metabolic syndrome, and hyper-leptinemia and leptin resistance due to obesity can have detrimental effects on the β-cell, contributing to impaired glucose stimulated insulin secretion [49] and proliferative capacity [50]. Experiments ex vivo using human and rodent islets, and in vivo using rodent models have shown dramatic impairments in insulin secretion with exposure to leptin [51]. Acting through suppressor of cytokine signalling 3 (SOCS3), leptin has been shown to impact insulin transcription in pancreatic islets [51], as well as the brain [52]. Conversely, the adipokine adiponectin decreases with obesity and metabolic dysfunction, and is generally known to potentiate insulin sensitivity and promote energy homeostasis [53]. In pregnancy, levels of circulating adiponectin decrease; however, individuals who are at risk for GDM have shown markedly reduced circulating adiponectin early in gestation, and dysregulated adiponectin molecular weight distribution in the circulation [54,55].

At the level of the β-cell, adiponectin is associated with increased proliferation and expansion although not necessarily with increased glucose stimulated insulin secretion [53,55] (Figure 2). Qiao et al. determined that, in pregnancy, adiponectin-knockout mice were not necessarily more insulin resistant, rather insulin insufficient; markers of insulin sensitivity were not markedly altered in adiponectin-knockout dams, but serum insulin and glucose-stimulated insulin secretion were reduced, and β-cell were significantly smaller than wild-type dams [53]. This suggests adiponectin signalling may play an important role in β-cell adaptations required in pregnancy. Adiponectin may also help protect against the lipotoxic damage of β-cells, and mediate inflammatory cytokine secretion from adipose tissue [56].

## 9. Placental Signalling and Metabolism

While technically a fetal organ, the placenta acts as an important interface between maternal and fetal environments and has been shown to respond in a variety of ways to maternal stress including gestational diabetes. Throughout gestation, ratios of placental lactogens will change in order to promote fetal growth and maintain metabolic homeostasis; ratios of placental lactogen (PL) to variant growth hormone (GH-V) have been postulated to predict whether the maternal environment is favoring β-cell adaptations required for healthy glucose metabolism in pregnancy [57]. This could represent another way in which placental signalling impacts maternal metabolism [57]. It has also been suggested that, in pregnancies affected by GDM, downstream mediators of insulin signalling such as mTOR and insulin-like growth factor (IGF) pathways are altered, with the end result being increases in nutrient transporters [17,58]. Increased amino acid, glucose, and lipid transporters on the placenta are associated with increased fetal growth and large for gestational age (LGA) infants; however, it is unclear whether these effects are due to GDM or treatment, which frequently includes insulin [59]. Additionally, when the fetus receives excess glucose due to maternal hyperglycemia (and corresponding increases in glucose transport), the elevated insulin production by maternal/fetal islets can accelerate fetal growth [60]. Conversely, placental transport of DHA (crucial for development of the fetal brain) is seen to be impaired in GDM although the implications of this are so far unclear [60]. 

Adiponectin has been shown to be expressed and secreted by rodent and human placenta, and the effect of adiponectin on the fetus appears to counter the effects of insulin: decreasing insulin signalling and slowing fetal growth [59]. Not surprisingly, the expression and secretion of adiponectin and its receptors by the human placenta is altered in the presence of GDM and can be modulated by cytokines associated with inflammation and metabolic stress (e.g., IFNy, TNFa, IL6, Leptin). In this vein, placental adiponectin may also play an important role in mediating insulin signalling to the fetal environment and impacting fetal growth [17,61]. 

In the context of the pancreatic β-cell, recent research has identified putative pathways for placental crosstalk with the islet in mice [62]. More specifically, ligands for G-protein coupled receptors that, independent of lactogens, are part of the placental secretome and may be involved in pregnancy-induced adaptive expansion in islets [62]. Corticotrophin releasing hormone (CRH) receptor was shown to be elevated mid-gestation in islets, and CRH expression was increased at the same time in the placenta; CRH has been shown to mediate insulin secretion [63] and β-cell proliferation [64] and could be an important mediator of β-cell adaptation potentially stimulated by placental signalling.

## 10. Current Therapeutic Strategies

Therapeutic approaches for GDM, while similar to those for type 2 diabetes, are complicated by the added complexity of pregnancy. Ideally, hyperglycemia in pregnancy can be controlled with lifestyle interventions including diet and physical activity, and appropriately limited gestational weight gain for women who are overweight (15–25 lbs) or obese (10–20 lbs) [65]. Some research has shown that, although obese women with GDM may gain less weight in pregnancy, they are more likely to require insulin treatment, and therapy is initiated earlier in pregnancy [66]. Without pre-existing obesity, lean women with GDM have been reported to have higher levels of insulin resistance and more pronounced β-cell insufficiency [67]. Although statistics vary with the population studied, up to 80% of women can be successfully treated using lifestyle interventions alone [65]; however, there are difficulties with adherence to lifestyle based interventions, and much of the available information relies on self-reported data. Even when trials of dietary interventions report efficacy, there is little consensus on ideal dietary composition, and adherence statistics vary widely [68]. This highlights a caveat of lifestyle and dietary interventions—implementing effective, healthy changes can be challenging and with the added stress of pregnancy and anxiety accompanying diagnosis, the risk of non-compliance to these recommendations is high [69]. In order to prevent maternal complications and limit exposure of the developing fetus to hyperglycemia and its long-term implications on health, pharmacological intervention is occasionally necessary [65]. 

It is important to consider metabolic changes in pregnancy, and the ability of pharmacologic agents to cross the placenta and any potentially teratogenic effects. Traditionally, insulin therapy is initiated if glycemic targets are not met using lifestyle interventions alone [70]. Although some studies have reported larger infants resulting from pregnancies treated with insulin compared to other oral antihyperglycemic agents (OAA), and an increased risk of neonatal hypoglycemia, it is generally accepted that the risks associated with treatment with currently approved medications during pregnancy are lower than the risks of untreated GDM for adverse health outcomes [70,71]. Additionally, therapeutic efficacies of different forms of insulin versus OAA are comparable and choice should largely be determined on a patient-to-patient basis. Some studies suggest lower risk of neonatal hypoglycemia with use of metformin (an insulin sensitizing biguanide) in comparison to insulin therapy; however, due to increased metabolism in pregnancy, higher doses may be required [72,73].

Sulfonylureas such as glyburide are considered insulin secretagogues, which potentiate the release of insulin through binding of the potassium/ATP channel on the β-cell. Glyburide use has been approved in pregnancy; however, studies have shown some elevated risk of neonatal hypoglycemia compared to insulin, and increased expression of GLUT1 on the placenta which may facilitate higher nutrient transfer to the fetus and confer higher risk of macrosomia [73,74]. In Canada, insulin is considered to be the first line approach, followed by metformin or glyburide [73].

While available therapeutics represent valuable tools for managing GDM, they do come with their own unique risks and contraindications. As pregnancy is recognized as a period of metabolic plasticity, particularly within the context of the endocrine pancreas, it also represents a unique opportunity to exploit those mechanisms in the context of therapeutic intervention. Currently, sulfonylureas constitute the only therapies targeted to the β-cell that are approved for use in GDM; however, mechanistic studies and large-scale –omics approaches could identify additional pathways and potential mediators of β-cell adaptation and failure that could be therapeutically targeted.

## 11. β-Cell Targeted Therapies for GDM

Many studies have identified inflammation and oxidative stress as key mediators of both insulin resistance and β-cell failure. A case-controlled study of anti-oxidants or anti-inflammatories (either as supplements or dietary sources) as adjunct therapies may improve outcomes [75]. With the body of evidence implicating lipotoxicity in β-cell failure, approaches that reduce circulating lipids in GDM may potentiate glycemic control and improve risks of complications; however, current pharmacological therapies for hyperlipidemia and hypercholesterolemia are not widely studied in pregnancy [76]. There is a precedent for use of fibrates such as gemfibrozil in cases of severe hypertriglyceridemia in pregnancy, with no teratogenicity if used after the 1st trimester, but are initiated only after implementation of dietary intervention and supplements have failed [77]. Statins for treatment of hypercholesterolemia are contraindicated in pregnancy; however, the argument has been made that teratogenicity observed in animal studies may be due to the dosages being significantly higher than what is therapeutically used in humans [78]. Supplements such as Omega-3 fatty acids and niacin have been shown to improve triglyceride and cholesterol levels in pregnancy, but studies are scarce and there is no consensus regarding dosage [76]. Studies in rodent models of diabetic pregnancy using resveratrol therapy have noted improved outcomes in terms of inflammation, glycemic control and circulating lipids, but no controlled studies in pregnant women currently exist [79]. 

Identification of downstream mediators of β-cell adaptations, as well as points at which failure and decompensation, can occur, lays the necessary groundwork for future therapeutic strategies including drug and nutritional interventions. The characterization of miRNA regulation allows for another layer of complexity, and potentially a target for the fine-tuning of β-cell function. Additionally, research into β-cell adaptations can help identify risk factors and preventative strategies for GDM—for example, understanding the diabetogenic effects of medications affecting serotonin signalling [7]. In vitro drugs that agonize the htr3 receptor and affect serotonin signalling in the β-cell can improve glucose stimulated insulin secretion, illustrating some therapeutic potential [28]. Currently, rodent models of GDM have shown that supplementation with adiponectin can improve glycemic control and insulin resistance [53,80]; however, this hasn’t been established in human pregnancy despite research showing a correlation between hypo-adiponectinemia and GDM [81].

## 12. The Future of β-Cell Targeted Therapeutics

While outright replacement therapies—using cadaveric islets [82] or stem cells [83]—are a promising proof of concept, the scarcity of donor islets and the level of invasiveness related to stem-cell procedures limit its potential. Mesenchymal stem cell (MSC) therapy for type-2 diabetes has been shown to stimulate β-cell replication, differentiate into insulin producing cells as well as play a protective role within the endocrine pancreas [84], and studies in rodents have shown the ability to improve symptoms in streptozotocin-induced diabetic rats [85]. Human clinical trials are ongoing; however, there are concerns regarding duration of efficacy, and no consensus has been reached regarding the ideal method of administration or primary measure of outcomes [84]. As of 2018, Canadian trials involving lab grown stem cells are only performed in individuals with a high-risk form of type-1 diabetes [83]; the transient nature of GDM and the invasiveness of these procedures make it unlikely to ever be considered for treatment of GDM. Regardless of direct therapeutic potential, the mechanistic insights gained from studies in rodents [85,86] and humans (particularly using MSC) could provide valuable information as to potential targets for pharmacological intervention. 

Ongoing research has also shown that, during periods of metabolic stress, insulin producing cells can be differentiated from non-β-cells. While neogenesis typically occurs from stem or progenitor cells [16], transdifferentiation, in which cells were already differentiated into a different cell type, can be triggered to differentiate into β-cells [87]. Acinar and duct cells within the pancreas in both human and rodent models have demonstrated the ability to transdifferentiate into insulin-producing cells [87,88]. In pregnant mice, there is evidence to suggest that non-β-cell progenitors contribute to the observed increase in β-cell mass [89]. The ability to trigger differentiation of progenitor and non-β-cells into insulin producing cells, even in vitro, provides an additional avenue for treatment options, either pharmacologically (by triggering entry into cell cycle [90] or with adenoskine kinase inhibitors [91] or replacement therapies [92]. 

## 13. Conclusions

It is apparent that the adaptive and compensatory response of the pancreatic β-cell to pregnancy is a growing area of interest; a number of very comprehensive studies have characterized many of the mechanisms involved. Increasing research in the area using large scale –omics approaches, cohort studies, animal models and stem cells are elucidating the complex pathways involved in β-cell adaptations in pregnancy. The putative targets that are emerging could yield novel therapeutic strategies that are not only beneficial for GDM therapy but potentially also for type 1 and 2 diabetes. Just as important as intervention is identification of predictive markers and risk factors that allow early monitoring and selection of the most effective therapeutic strategies for women with GDM (e.g., individuals who respond to lifestyle therapy vs. individuals that would benefit from immediate insulin or OAA therapy). While maternal insulin resistance is crucial for fetal growth, the ability of the β-cell to compensate for this necessary phenomenon may be the difference between a healthy pregnancy and GDM.
